# Farnesyltransferase inhibitor treatment restores chromosome territory positions and active chromosome dynamics in Hutchinson-Gilford progeria syndrome cells

**DOI:** 10.1186/gb-2011-12-8-r74

**Published:** 2011-08-12

**Authors:** Ishita S Mehta, Christopher H Eskiw, Halime D Arican, Ian R Kill, Joanna M Bridger

**Affiliations:** 1 Progeria Research Team, Centre for Cell and Chromosome Biology, Biosciences, School of Health Sciences and Social Care, Kingston Lane, Brunel University, West London, UB8 3PH, UK; 2 Current address: B-202, Department of Biological Sciences, Tata Institute of Fundamental Research, Homi Bhabha Road, Mumbai-400005, India

## Abstract

**Background:**

Hutchinson-Gilford progeria syndrome (HGPS) is a premature ageing syndrome that affects children leading to premature death, usually from heart infarction or strokes, making this syndrome similar to normative ageing. HGPS is commonly caused by a mutation in the A-type lamin gene, *LMNA *(G608G). This leads to the expression of an aberrant truncated lamin A protein, progerin. Progerin cannot be processed as wild-type pre-lamin A and remains farnesylated, leading to its aberrant behavior during interphase and mitosis. Farnesyltransferase inhibitors prevent the accumulation of farnesylated progerin, producing a less toxic protein.

**Results:**

We have found that in proliferating fibroblasts derived from HGPS patients the nuclear location of interphase chromosomes differs from control proliferating cells and mimics that of control quiescent fibroblasts, with smaller chromosomes toward the nuclear interior and larger chromosomes toward the nuclear periphery. For this study we have treated HGPS fibroblasts with farnesyltransferase inhibitors and analyzed the nuclear location of individual chromosome territories. We have found that after exposure to farnesyltransferase inhibitors mis-localized chromosome territories were restored to a nuclear position akin to chromosomes in proliferating control cells. Furthermore, not only has this treatment afforded chromosomes to be repositioned but has also restored the machinery that controls their rapid movement upon serum removal. This machinery contains nuclear myosin 1β, whose distribution is also restored after farnesyltransferase inhibitor treatment of HGPS cells.

**Conclusions:**

This study not only progresses the understanding of genome behavior in HGPS cells but demonstrates that interphase chromosome movement requires processed lamin A.

## Background

Hutchinson-Gilford progeria syndrome (HGPS) is an extremely rare disorder that affects children causing them to age prematurely [1]. Clinical features of this disease include alopecia, growth retardation, an extremely aged appearance, loss of subcutaneous fat, progressive atherosclerosis, bone deformaties and cardiovascular diseases [2-5]. HGPS is most frequently caused by an autosomal dominant *de novo *mutation in the *LMNA *gene, which encodes the nuclear intermediate filament proteins lamin A and lamin C [6]. These A-type lamins are both components of the nuclear lamina at the inner nuclear envelope and of the nuclear matrix [7-10]. Lamin proteins have roles in DNA replication, transcription, chromatin organization, maintenance of nuclear shape and integrity and in cell division [11,12]. The most common mutation associated with HGPS is a single base substitution in codon 608 of exon 11 on the *LMNA *gene resulting in the formation of a cryptic splice site that produces a truncated pre-lamin A protein called progerin, lacking 50 amino acids near the carboxyl terminus [6,13]. Progerin acts in a dominant negative manner on the nuclear functions of cell types that express lamin A, which comprise the majority of differentiated cells derived from mesenchymal stem cells [14].

In normal cells, pre-lamin A contains a CaaX motif at the carboxy-terminal end, where the cysteine residue becomes farnesylated by the enzyme farnesyltransferase [15]. The presence of a farnesyl group at the carboxy-terminal end, along with the CaaX motif, promotes the association of pre-lamin A with the nuclear membrane and these are thus vital for correct localization of the mature protein [16]. The protein undergoes an endo-proteolytic cleavage by the enzyme ZMPSTE24-FACE1 metalloproteinase [17], resulting in the cleavage of 15 amino acids at the carboxy-terminal end, including the farnesylated cysteine, producing mature lamin A [18]. In HGPS, an activation of the cryptic splice site results in an internal deletion of 50 amino acids near the carboxy-terminal end of the protein, including the ZMPSTE24-FACE1 cleavage site. This deletion does not affect the CaaX motif and the progerin undergoes normal farnesylation, but it lacks the ZMPSTE24-FACE1 recognition site necessary for the final cleavage step and hence remains farnesylated [13,19]. Retention of the farnesyl group and accumulation of the farnesylated protein at the nuclear envelope compromises nuclear integrity and leads to formation of abnormally shaped nuclei, a prominent characteristic seen in HGPS [20,21]. The concept that blocking the farnesylation of progerin might help ameliorate disease pathology seen in HGPS cells was proposed in 2003, shortly after the discovery of the gene involved in causing HGPS. Thus, drugs called farnesyltransferase inhibitors (FTIs), which inhibit attachment of a farnesyl group to a protein by irreversibly binding to the CaaX domain [22], were used in both *in vitro *and *in vivo *analyzes. The lack of a progeria phenotype in a knock-in mouse model expressing non-farnesylatable progerin supports this approach [23].

*In vitro *studies have demonstrated that treating HGPS cells with FTIs prevents the accumulation of progerin at the nuclear envelope and reduces the frequency of abnormally shaped nuclei in culture [3,24-27], reduces nuclear blebbing as well as the redistribution of mutant protein from the nuclear envelope [3], and restores genome localization after mitosis [28] and the distribution of nucleolar proteins [29]. HGPS cells treated with FTIs for 72 hours also showed improved nuclear stiffness to levels almost comparable to normal cells and significant restoration of directional persistence with regards to cell migration and thus improvement in wound healing ability [30]. Another study demonstrated that double strand break repair was improved in HGPS cells after FTI treatment [31]. Treatment with FTIs has also been employed in animal models with positive results. FTI treatment of ZMPSTE24-/- mice resulted in the presence of non-farnesylated prelamin A, improved growth curves, bone integrity and body weight [19], and a reduction of rib fractures [27,32-34]. The study in *Lmna *HG/+ mice demonstrated that FTI treatment improved body weight and bone structure, with improvement in bone mineralization and cortical thickness [32]. A more recent study that uses a transgenic mouse model carrying the human G608G *LMNA *mutation and displaying a cardiovascular phenotype demonstrated that FTI treatment reduces vascular smooth muscle cell loss and proteoglycan accumulation and thus prevented the onset as well as the progression of cardiovascular diseases in these mice [35].

One of the shortcomings that FTI treatment has been confronted with is that these drugs may cause an alternative post-translational modification of pre-lamin A or progerin [36]. Pre-lamin A and progerin are both geranylgeranylated by the enzyme geranylgeranyltransferase when they are not permitted to undergo farnesylation in the presence of FTIs [37]. Inhibition of both enzymes, that is, farnesyltransferase and geranylgeranyltransferase, using a FTI and a geranylgeranyltransferase inhibitor (GGTI) simultaneously results in accumulation of substantially higher levels of normal pre-lamin A [37]. Thus, in the present study we have used both types of drugs, FTIs and GGTIs, to inhibit progerin processing *in vitro*.

Interphase chromosome territories are positioned non-randomly in a radial pattern in nuclei, with gene-rich chromosomes located towards the nuclear interior, gene-poor chromosomes towards the nuclear periphery and chromosomes carrying intermediate gene loads in an intermediate position [38,39]. It has been demonstrated that chromosome position is altered in cells that leave the cell cycle reversibly into quiescence or irreversibly into senescence [40-43] (IS Mehta, KJ Meaburn, M Figgitt, IR Kill, JM Bridger, manuscript in preparation). In addition, we have previously shown that interphase radial chromosome positioning is altered in the nuclei of proliferating human dermal fibroblasts (HDFs) derived from patients diagnosed with different laminopathies, including classical HGPS [41]. We revealed that chromosomes 13 and 18, normally located at the nuclear periphery in unaffected proliferating HDFs, are found in the nuclear interior in proliferating laminopathy cells, mimicking their position in non-proliferating control cells [41,43]. One other study has observed mis-localization of chromosome 13 in cells from a patient with a E161K mutation in *LMNA *[44]. Others have also shown that heterochromatin is disorganized in HGPS cells [20,45,46], implying that lamin A is important in chromatin organization and chromosome territory location in interphase nuclei, both of which are perturbed in laminopathy cells. Furthermore, we have recently demonstrated that normal human primary fibroblasts respond to removal of serum by rapidly repositioning specific chromosomes within interphase nuclei and that this movement requires nuclear myosin 1β (NM1β) [42]. NM1β is now being considered as a component of a nuclear motor system that can move chromatin around interphase nuclei [47-50]. It has also been found to be a lamin A binding partner [51].

In this study we have analyzed chromosome positioning in nuclei derived from primary HGPS fibroblasts and found that chromosome positioning in proliferating HGPS cells mimics that of control quiescent (serum-starved) fibroblasts. By treating cells *in vitro *with a FTI alone and in combination with a GGTI we re-established a nuclear distribution of specific chromosomes in proliferating HGPS cells that is found in control proliferating fibroblasts. The treatment also restored the response to serum removal in the cell population so that chromosomes became relocated within 15 minutes of serum removal, as they would in control cells. Furthermore, we found that the nuclear distribution of NM1β was aberrant in proliferating HGPS cells but after FTI treatment it was redistributed and restored to a similar distribution as seen in control proliferating fibroblasts. Thus, in HGPS cells FTI treatment restores normal chromosome positioning, the rapid relocation of whole chromosomes in response to low serum and the distribution of NM1β. Therefore, by preventing the farnesylation of progerin in HGPS cells, chromosomes behave correctly, possibly due to the correct organization of NM1β. This indicates that lamin A is involved in regulation of chromosome behavior through a nuclear motor structure.

## Results

### Interphase chromosome locations in HGPS fibroblast nuclei resemble those of quiescent (serum-starved) control fibroblasts

We determined the radial positions of three representative chromosomes in interphase nuclei of HGPS cells; chromosomes 10, 18 and X. Chromosome 10 is found in different nuclear positions in proliferating, quiescent and senescent nuclei [42,43]. Chromosome 18 moves from the nuclear periphery to the interior when cells transit from proliferation to a non-proliferative state and is found in the nuclear interior in proliferating laminopathy cells, including an HGPS cell line [41]. The X chromosome remains at the nuclear periphery in all cell cycle states and is located at the periphery in all laminopathy cells analyzed [41] and as such is used as a negative control for chromosome repositioning.

To position chromosomes by fluorescence *in situ *hybridization (FISH) in interphase nuclei, we fixed cells in methanol:acetic acid (3:1) to produce flattened cytoplasm-free nuclei followed by two-dimensional FISH with specific chromosome paints. More than 50 digital images were then used in an erosion analysis that creates five concentric shells of equal area across the nucleus and the amount of DNA signal (DAPI) and chromosome paint signal were measured in each shell [38,39]. To normalize the data, fluorescence intensity of the chromosome signal was divided by the intensity of the DNA signal and the data were plotted as histograms, with the nuclear periphery represented by shell 1 and the nuclear interior by shell 5. The proliferative status of the cells is determined by indirect immunofluorescence using antibodies to the proliferative marker Ki-67 [52]. Positive signal indicates that the cells are in proliferative interphase whereas cells negative for Ki-67 in cultures kept in high serum denote senescent cells [53]. Young quiescent cells, that is, serum starved or cells that have reached confluency, are also negative for anti-Ki-67.

**Figure 1:**
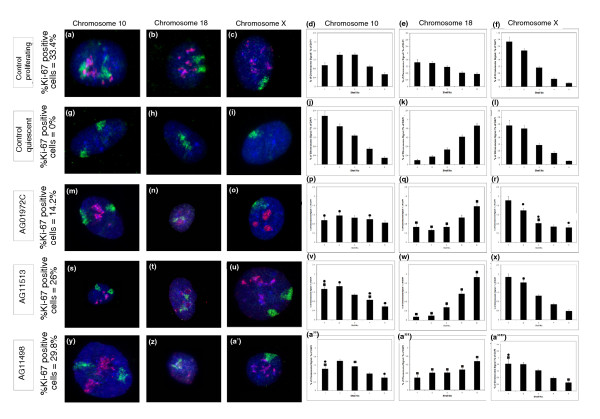
**Positions of chromosomes 10, 18 and × in HGPS human dermal fibroblasts**. HDFs derived from HGPS patients were subjected to two-dimensional FISH in order to delineate and analyze the nuclear positions of chromosome 10, 18 and × territories. **(a-c, g-i, m-o, s-u, y-a') **Chromosome territories are shown in green, pKi-67, a proliferation marker, is shown in red and DAPI staining in blue delineating the nuclear DNA. Scale bar = 10 μm. **(d-f, j-l, p-r, v-x, a'-a''') **Histograms displaying the distribution of chromosome signal for each chromosome analyzed using erosion analysis. Bars represent percentage mean normalized proportion of chromosome signal for each chromosome, with shell 1 being located at the nuclear periphery and shell 5 the nuclear interior. Error bars represent standard error of the mean. In control proliferating fibroblasts chromosome 10 occupies an intermediate nuclear location (a, d) and chromosomes 18 (b, e) and × (c, f) a location at the nuclear periphery (b, e). In control fibroblasts made quiescent by serum starvation both chromosomes 10 (g, j) and × (i, l) are located at the nuclear periphery. In the HGPS cells, chromosome 10 (m, p, s, v, y, a'') and chromosome × (i, l, o, r, u, x, a'''') also occupy a peripheral location in nuclei whilst chromosome 18 (n, q, t, w, z, a''') is positioned in the nuclear interior. Unpaired, unequal variances two-tailed students *t*-tests were performed to assess statistical differences between the position of chromosome territories in proliferating HGPS fibroblasts and that of control proliferating and quiescent control cells. Filled-in squares indicate a difference when compared to control proliferating HDFs, filled in circles indicate a difference when compared to control quiescent HDFs (*P *< 0.05).

Figure 1a, d confirms that chromosome 10 occupies an intermediate location in proliferating control nuclei (as determined by pKi-67 staining) and a peripheral location in control quiescent nuclei (Figure 1g, j). Figure 1p, v, a'' reveals that chromosome 10 is located at or towards the nuclear periphery in proliferating HGPS nuclei. Chromosome 18 is located towards the nuclear periphery in proliferating control cells (Figure 1e) but is then interior in control quiescent cells (Figure 1k), and in all three HGPS cell lines (Figure 1q, w, a'''). Chromosome × is found at the nuclear periphery in control proliferating (Figure 1f) and quiescent cells (Figure 1l), as well as in all three HGPS cell lines (Figure 1r, x, a''''). These relative positions for chromosomes 10 and × have been confirmed using three-dimensional fixation, laser scanning confocal microscopy, optical image reconstruction and measurement in three-dimensions (Figure S1 in Additional file 1).

**Figure 2:**
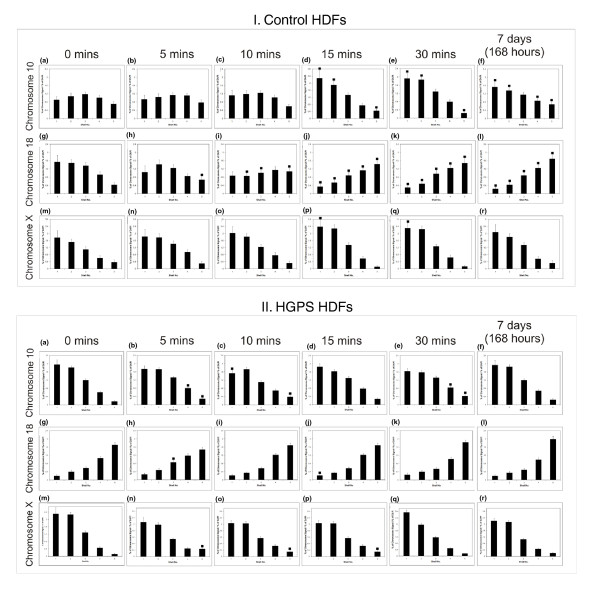
**Positions of chromosomes 10, 18 and × in HGPS human dermal fibroblasts following serum starvation Proliferating control and HGPS (AG11498) cells were placed in low serum (0.5%) for 5 minutes, 10 minutes, 15 minutes, 30 minutes and 7 days**. The cells were subjected to two-dimensional FISH and the nuclear location of chromosome 10 **(I:a-f, II:a-f,)**, chromosome 18 **(I:g-l, II:g-l,) **and the X chromosome **(I:m-r, II:m-r,) **assessed by erosion analysis. Unpaired, unequal variances two-tailed students *t*-tests were performed to assess statistical differences between the fibroblasts at 0 minutes and the cells placed in low serum. The filled-in squares represent a significant difference (*P *< 0.05). Panel I demonstrates that when control fibroblasts are placed in low serum, chromosome 10 moves from an intermediate position to a peripheral position (I:a-f), chromosome 18 moves from a peripheral position to an interior location (I:g-l) and chromosome × remains at the nuclear periphery (I:m-r). These changes in position have been revealed previously [40,42].

We have recently shown that chromosomes relocate very rapidly to new nuclear locations in control proliferating fibroblasts placed into low serum [42]. When proliferating control fibroblasts (Figure 2a) are placed in low serum, chromosome 10 moves towards the nuclear periphery within 15 minutes (Figure 2I:d), chromosome 18 repositions from the nuclear periphery in proliferating fibroblasts (Figure 2I:g) to the nuclear interior, again within 15 minutes of incubation in low serum medium (Figure 2I:j), and chromosome × remains at the nuclear periphery from 0 minutes to 7 days (Figure 2I:m-r). When HGPS cells (AG11498) are placed in low serum there is no significant change in chromosome location over 7 days; that is, chromosome 10 remains near the nuclear periphery (Figure 2II:a-f), chromosome 18 remains in the nuclear interior (Figure 2II:g-l) and chromosome × remains at the nuclear periphery (Figure 2II:m-r).

### FTI treatment restores wild-type interphase chromosome positions in HGPS cells for at least two passages

**Figure 3:**
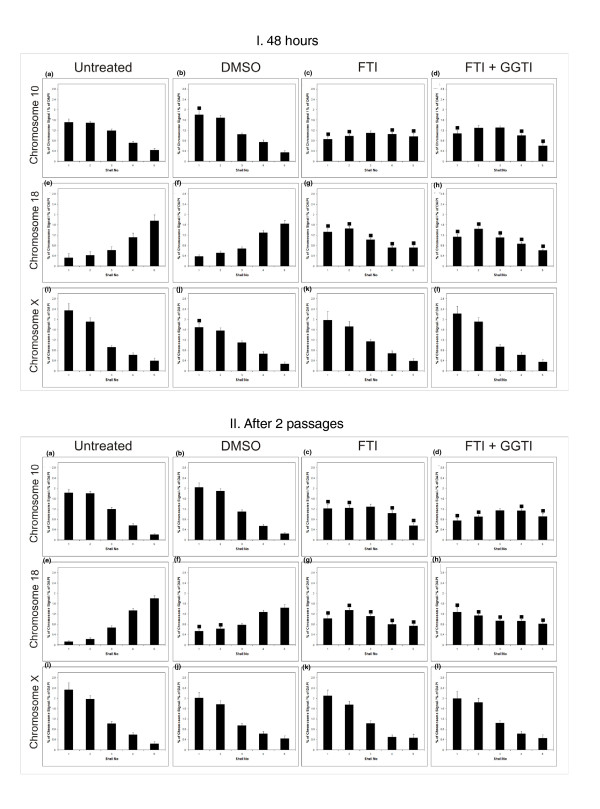
**Positions of chromosomes 10, 18 and × in HGPS human dermal fibroblasts after FTI treatment (48 hours) and after two passages**. Histograms displaying the normalized nuclear positions of chromosome 10, 18 and × territories in interphase nuclei subjected to two-dimensional FISH analysis, and determined by erosion analysis, of untreated AG11498 HGPS fibroblasts **(I:a, f, k)**, AG11498 HDFs treated with equivalent amounts of DMSO used for dissolution of inhibitors **(I:b, g, l)**, AG11498 fibroblasts treated with 2.5 μM FTI-277 **(I:c, h, m)**, AG11498 fibroblasts treated with 2.5 μM GGTI-2147 **(I:d, i, n) **and AG11498 HDFs treated with a combination of FTI-277 and GGTI-2147 (2.5 μM each) **(I:e, j, o)**. Shell 1 represents the nuclear periphery and shell 5 the nuclear interior. Error bars indicate the standard error of the mean. Filled-in squares indicate statistical difference (*P *< 0.05) for that shell when compared to the equivalent shell of the untreated sample. Panel II displays the nuclear positioning of cells treated with the inhibitors for 48 hours and then cultured for a further two passages.

FTIs have been used to correct several cellular aberrations in HGPS cells and in whole organisms. It has been suggested that by blocking farnesylation, certain proteins can be alternatively modified by geranylgeranylation. Thus, we employed FTI-277 both separately and simultaneously with GGTI-2147 to determine if we could restore chromosome position to normal in HGPS cells. An HGPS cell line (AG11498) was treated with 2.5 μM FTI-277 (Figure 3I:c, g, k) and with 2.5 μM each of FTI-277 and GGTI together (Figure 3I:d, h, l). The small amount of DMSO that was used to dissolve the inhibitors was used as a control (Figure 3I:b, f, j). As expected, the X chromosome did not change nuclear position with any of the treatments. However, with FTI-277 alone and together with GGTI-2147, chromosome 10 became located in an intermediate radial location in nuclei (Figure 3I:c, d). Chromosome 18 was also repositioned after treatment with FTI-277 alone and together with GGTI-2147 from an internal location to a peripheral one (Figure 3I:g, h). Chromosome × was not repositioned after FTI-277 treatment alone nor with FTI-277 and GGTI-2147 together (Figure 3I:k, l). DMSO alone had no significant effect on chromosome repositioning (Figure 3I:b, f, j).

After the 48 hour treatments the inhibitors were removed and the cells permitted to go through two more passages before chromosome positioning was analyzed again (Figure 3II). The newly corrected chromosome positions in the HGPS cells were maintained for treatment with FTI-277 alone and FTI-277 together with GGTI-2147.

### Chromosomes are rapidly repositioned in FTI-treated HGPS cells responding to low serum

**Figure 4:**
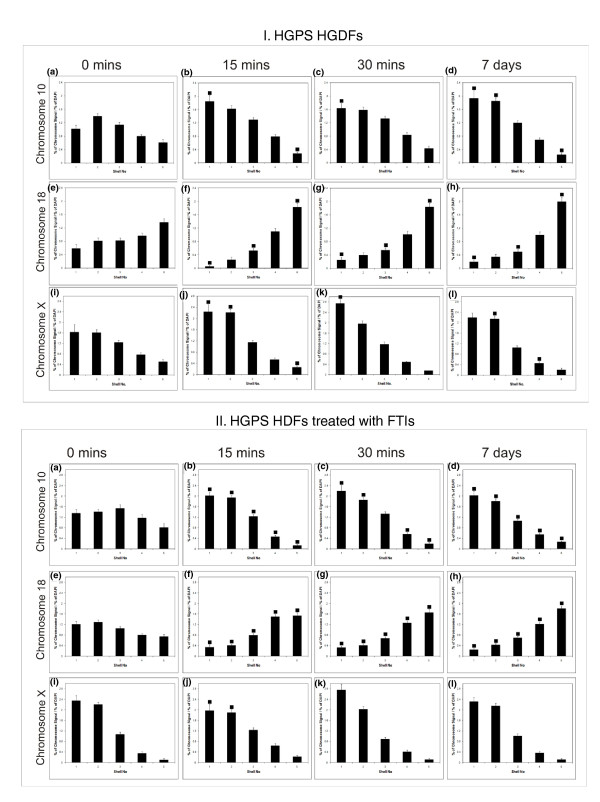
**Positions of chromosomes 10, 18 and × in HGPS human dermal fibroblasts after FTI treatment following incubation in low serum**. Control fibroblasts and HGPS (AG11513) cells treated with both FTI-277 and GGTI-2147 were placed in low serum, fixed and subjected to two-dimensional FISH and erosion analysis, with shell 1 representing the nuclear periphery and shell 5 the nuclear interior. The histograms display the positions of chromosomes 10, 18 and × in interphase nuclei after 0 **(a, e, i)**, 15 **(b, f, j)**, 30 **(c, g, k) **minutes and 7 days **(d, h, l) **following serum withdrawal. Error bars indicate the standard error of the mean. Filled-in squares indicate statistical difference (*P *< 0.05) for that shell when compared to the equivalent shell for 0 minute sample. The nuclear repositioning of chromosomes 10 and 18 after serum removal [42] was restored in the FTI-treated HGPS cells.

After the HGPS cells had been treated with FTI-277 we wished to see if the rapid active chromosome repositioning after serum removal [42] was restored. Indeed, for all three chromosomes the starting location in proliferating nuclei was similar to that in the control cells and movement from an intermediate location to the periphery and the periphery to interior for chromosomes 10 and 18, respectively, was apparent after just 15 minutes (Figure 4II:b, f) with no change in the nuclear position of the X chromosome (Figure 4II:j). The shape of any aberrant, herniated, invaginated nuclei was also restored to more smoothened ellipsoid shapes after the 48-hour treatment with FTI-277 (data not shown).

### Distribution of NM1β in progeria cells before and after FTI treatment

**Figure 5:**
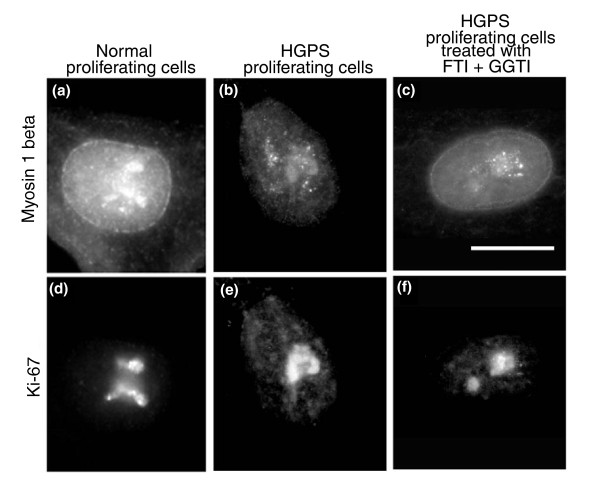
**Nuclear myosin 1β staining in control fibroblasts and HGPS fibroblasts before and after FTI treatment**. NM1**β **is a component of the nuclear motor complex that is involved in chromosome movement. Control and HGPS (AG11498) fibroblasts (untreated and treated with FTI and GGTI together) were fixed with 1:1 methanol:acetone and subjected to dual indirect immunofluorescence with commercial rabbit anti-NM1β antibody **(a-c) **and a mouse anti-pKi67 (to reveal proliferating cells) **(d-f)**. Secondary antibodies were a donkey anti-mouse antibody conjugated to FITC and a swine anti-rabbit antibody conjugated to TRITC. (a) In proliferating control fibroblasts the distribution of NM1β is at the nuclear periphery, in the nucleolus and throughout the nucleoplasm in a fine punctate distribution. (b) In proliferating HGPS fibroblasts slight nucleoplasmic anti-NM1β staining is apparent but staining is mainly distributed as large aggregates near the nucleoli. (c) When HGPS cells were treated with FTI and GGTI together, the distribution of NM1β was restored to the nuclear periphery and throughout the nucleoplasm, although there were still large aggregates present. Scale bar = 10 **μ**m.

There is evidence that rapid chromosome repositioning using the serum removal assay is elicited through nuclear motor activity, probably involving NM1β [42]. We used an antibody to NM1β that we and others have employed previously [42], and analyzed the nuclear distribution of this protein in HGPS cells. In control fibroblasts, NM1β is distributed homogenously or as fine punctuate foci throughout the nucleoplasm with a concentration at the nuclear periphery and the nucleolus (Figure 5a) [42]. The distribution of NM1β in HGPS nuclei is very different to that found in control proliferating cells, being much more like the distribution observed in non-proliferating control cells [42]; there is some nucleolar anti-NM1β staining but, in addition, NM1β is localized in large aggregates towards the interior of the nucleus, without any localization at the nuclear periphery and some weak staining in the nucleoplasm (Figure 5b). Most of the proliferating HGPS cells displayed NM1β as aggregates (85.1%; Figure 5a, Table 1) but when they were treated with FTI-277 for 48 hours, 73.7% of them displayed a normal distribution of NM1β (Figure 5c, Table 1), while only 18.6% of treated HGPS cells displayed NM1β aggregates (Table 1).

**Table 1:** Distribution of NM1β in HGPS cells

	Proliferating HGPS cells	0 hours (quiescent HGPS cells)	24 hours	36 hours	48 hours
Nucleolar + rim + nucleoplasmic	5.8 ± 3.0	0.8 ± 2.0	2.7 ± 6.24	3.1 ± 3.06	10.4 ± 3.05
Nucleolar only	0 ± 0	19.8 ± 6.65	19.7 ± 6.506	8.0 ± 6.08	8.2 ± 3.05
Aggregates	85.1 ± 6.11	77.3 ± 6.55	71.9 ± 5.85	83.3 ± 3.06	76.4 ± 8.96
Rim + nucleoplasmic	5.2 ± 2.51	0 ± 0	2.2 ± 3.06	2.9 ± 4.0	2.2 ± 3.61
Nucleoplasmic	2.9 ± 2.64	1.4 ± 3.51	1.8 ± 3.51	1.8 ± 4.0	1.8 ± 2.64
Dull	0.1 ± 1.53	0.6 ± 0.57	1.7 ± 3.78	0.7 ± 2.0	0.9 ± 2.51

Proliferating HGPS cells were in 15% serum, quiescent HGPS cells were in 0.5% serum for 7 days, and 24, 36 and 48 hours refer to time after the re-addition of serum. The distribution of NM1β is normally nucleolar + rim + nucleoplasmic in proliferating control cells, while abnormal distributions are nucleolar only, aggregates, rim + nucleoplasmic, nucleoplasmic only and dull. In control quiescent cells aggregates are the most common distribution observed [42]. Numbers are percentage values ± standard deviation. The most prominent distribution of NM1β in HGPS cells in high and low serum was in aggregates.

**Figure 6:**
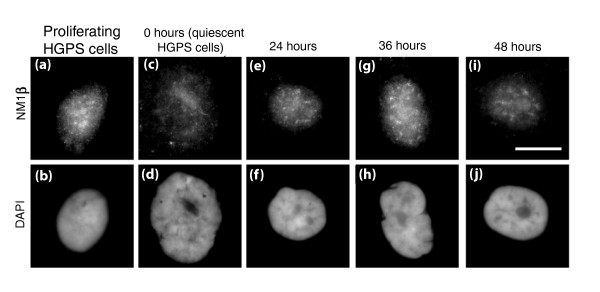
**Myosin staining pattern in quiescent HGPS human dermal fibroblasts following re-stimulation**. HDFs from HGPS patient AG11498 were serum-starved for 7 days to induce quiescence. The cells were then re-stimulated with fresh serum and samples were collected at 0, 24, 36 and 48 hours post-serum restoration. Samples were also collected before serum withdrawal (proliferating cells). The samples were then fixed with methanol:acetone (1:1) and distribution of NM1β was assessed by performing a dual color indirect immunofluorescence assay for NM1β **(a, c, e, g, i) **and pKi67 **(b, d, f, h, j)**. (a, c, e, g, i) The distribution of NM1β in cells before and after re-stimulation of quiescent fibroblasts. Scale bar = 10 μm.

**Figure 7:**
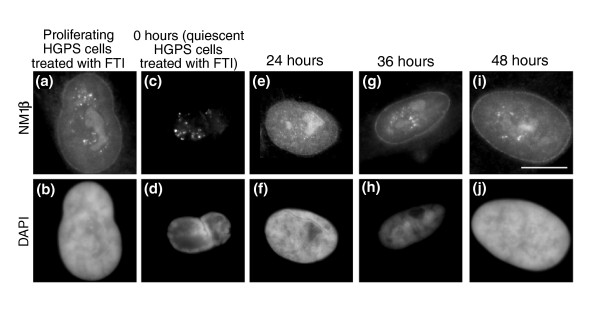
**Myosin staining pattern in quiescent HGPS human dermal fibroblasts after FTI treatment and following re-stimulation**. AG11498 HDFs treated with FTI-277 were serum starved for 7 days to induce quiescence. The cells were then re-stimulated with fresh serum and samples were collected at 0, 24, 36 and 48 hours after serum restoration. Samples were also collected before serum withdrawal (proliferating cells). The samples were then fixed with methanol:acetone (1:1) and distribution of NM1β was assessed by performing an indirect immunofluorescence assay for NM1β. **(a, c, e, g, i) **The distribution of NM1β in cells before and after restimulation of quiescent fibroblasts. **(b, d, f, h, j) **DAPI staining. Scale bar = 10 μm.

To determine if the NM1β distribution was different in HGPS cells that had been made quiescent, serum starved HGPS fibroblasts were also analyzed using indirect immunofluorescence with the anti-NM1β antibody. The distribution of NM1β in quiescent HGPS cells was similar to that in control cells made quiescent by serum starvation (Figure 6a), but also to that in proliferating HGPS cells (Figure 6c), which also had some aggregates of NM1β staining. In control cells that have been made quiescent and re-stimulated, the NM1β distribution returned to a proliferating-type distribution only 24 to 36 hours after the re-addition of serum [42]. Serum-starved HGPS cells (7 days) were re-stimulated with serum and samples taken at 24, 36 and 48 hours (Figure 6). In HGPS cells there was no significant difference in NM1β distribution (aggregates) in proliferating cells (85.1%; Figure 6a), in quiescent cells (77.3%; Figure 6c) or in cells re-stimulated with serum and fixed after 24 hours (71.9%; Figure 6e), 36 hours (83.3%; Figure 6g) and 48 hours (76.4%; Figure 6i). These data are given in Table 1 and demonstrate that the cells were not responding to growth factor cues with respect to NM1β, as occurs in control cells. However, if HGPS cells are treated with FTI and GGTI together for 48 hours, the distribution of NM1β becomes very similar to control cells, with more staining throughout the nucleoplasm and a concentration at the nuclear periphery and nucleolus (Figure 7a, Table 2). When the treated HGPS cells are made quiescent for 7 days, the distribution of NM1β in these cells is typical for a non-proliferating control culture, with large aggregates of NM1β. When quiescent cultures of HGPS cells treated with FTIs were re-stimulated by the re-addition of serum, the cells showed a more normal distribution of NM1β, with nucleoplasmic, nucleolar and nuclear rim staining. We observed increases in the normal distribution of NM1β from 2.1% in quiescent HGPS cells to 35% at 24 hours after re-stimulation (Figure 7e, Table 2), 51.6% at 36 hours (Figure 7g, Table 2) and 64% by 48 hours (Figure 7i, Table 2). This implies that the cells were able to respond to growth factors after the FTI treatment and re-position chromosomes using a nuclear motor activity that, in a further experiment, was blocked by the nuclear myosin inhibitor BDM (2,3-butanedione-2-monoxime; Figure S2 in Additional file 1), showing that we restored functional motor activity in HGPS cells for chromosome relocation.

**Table 2:** Distribution of NM1β in HGPS cells after a 48 hour FTI treatment

	Proliferating HGPS cells + FTI	0 hours (quiescent HGPS cells + FTI)	24 hours	36 hours	48 hours
Nucleolar + rim + nucleoplasmic	73.7 ± 7.09	2.1 ± 1.53	35.0 ± 8.18	51.6 ± 3.61	64.0 ± 4.0
Nucleolar only	0 ± 0	19.3 ± 11.32	14.9 ± 2.0	9.8 ± 3.46	8.5 ± 2.64
Aggregates	18.6 ± 6.55	74.2 ± 5.29	45.8 ± 1.53	31.5 ± 4.58	21.8 ± 2.51
Rim + nucleoplasmic	4.8 ± 2.08	0 ± 0	2.5 ± 1.53	3.8 ± 2.08	1.6 ± 0.06
Nucleoplasmic	1.8 ± 2.08	2.9 ± 2.08	1.2 ± 1.15	2.6 ± 3.21	2.1 ± 2.64
Dull	0.9 ± 1.53	1.5 ± 1.53	0.4 ± 1.73	0.5 ± 2.31	1.9 ± 3.79

Distribution was analyzed in FTI-treated HGPS cells in 15% serum (proliferating), 0.5% serum for 7 days (quiescent) and 24, 36 and 48 hours after the re-addition of serum. The distribution of NM1β in proliferating cells was more like control fibroblasts with a normal distribution of nucleolar + rim + nucleoplasmic that became like normal quiescent cells with the withdrawal of serum and returned to a more normal distribution for proliferating cells within the expected time frame (36 to 48 hours) [42]. Numbers are percentage values with ± standard deviation.

## Discussion

The HGPS cells in this study all have a cryptic splice site (G608G) that results in the accumulation of a toxic farnesylated lamin A termed progerin. We have previously shown that chromosome positioning is altered in a number of primary fibroblast lines derived from laminopathy patients [41], with the positioning of chromosomes 13 and 18 within the nuclear interior and not towards the nuclear periphery, as observed in control cells. The positioning of these chromosomes in proliferating laminopathy cells is similar to that in non-proliferating control cells, given that smaller chromosomes are found in the nuclear interior in the latter [41]. By examining the nuclear position of chromosome 10, which is located within different nuclear compartments in serum starved quiescent cells and senescent cells [42,43], we were able to determine that the position of chromosome 10 shown in this study in proliferating HGPS cells was as it would be in control quiescent cells. Goldman and colleagues [54] have shown that A-type lamins are involved intimately with genome organization since cells in which lamin B1 has been knocked down form nuclear blebs that specifically contain only A-type lamins. Interestingly, gene-rich regions of the genome are found in these blebbed areas, implying that changing the lamina structure and its properties directly affects genome behavior.

Recently, we demonstrated that chromosomes become relocated within interphase nuclei very rapidly after cells are placed in low serum [42]. We repeated this assay with HGPS cells. Since the chromosomes are already positioned in the nuclear locations they would be in in quiescent control cells, we recorded no significant change. We treated the HGPS cells with a FTI separately and in combination with a GGTI to preclude the inhibition of farnesylation being compensated for by the geranylation of the mutant lamin A. FTI treatment alone or in combination with GGTI resulted in both chromosomes 10 and 18 being relocated to the correct location as seen in control cells-that is, chromosome 10 in an intermediate location and chromosome 18 at the nuclear periphery. The chromosomes maintained these positions even after the HGPS cells had gone through two passages without the inhibitor. This reorganization of the genome means that chromosome territories have moved in various directions-for example, some moved away from the nuclear periphery whereas others moved towards it, possibly forming anchorage sites at the nuclear lamina [55].

We have already demonstrated that chromosome movement and relocation after serum removal is active, rapid and elicited through nuclear motor activity involving nuclear actin and myosins, such as NM1β [42]. Staining of proliferating HGPS cells with a commercial antibody against NM1β showed that NM1β was predominantly in large aggregates. However, when the HGPS cells were treated with a FTI alone or together with a GGTI, the nuclear distribution of NM1β became more like that in proliferating control cells, nucleoplasmic with prominent staining at the nucleolus and nuclear envelope. If NM1β is a component of a nuclear motor complex that is involved in moving chromosomes around, then its distribution and activity appear to be restored in HGPS cells treated with the FTI. This was confirmed in an experiment using BDM to block nuclear myosin activity in FTI-treated HGPS cells. After the BDM treatment chromosome 10 did not relocate to the nuclear periphery as it did in HGPS cells treated with the FTI alone. Nuclear motors are also involved in other nuclear activities, such as transcription and chromatin remodeling (reviewed in [56]), which may also be improved by FTI treatment in HGPS cells due to the reinstatement of NM1β. However, not be being able to move chromosomes around in the nucleus would have major implications for cellular differentiation and tissue regeneration in HGPS patients, since whole chromosomes and genes are moved and remodeled upon differentiation, correlating with gene expression [57-60]. We present here the hypothesis that global gene expression is affected in HGPS cells and that this will be restored upon normal chromosome localization. Furthermore, fully processed mature lamin A must be part of this dynamic process by either binding directly to nuclear motor proteins or by being part of a required nucleoskeleton [61] that provides support to the nuclear motor proteins.

## Conclusions

In this study we have demonstrated that proliferating HGPS cells have chromosome territory positions similar to quiescent control fibroblasts, as revealed by chromosome 10 painting. Using FTI/GGTI treatment to prevent progerin farnesylation and geranylgeranylation, we were able to restore normal interphase chromosome positioning. More importantly, this treatment restored the rapid relocalization of chromosomes following serum withdrawal. We already have evidence that chromosome movement requires NM1β [42]. Now we demonstrate that NM1β is distributed aberrantly in proliferating HGPS cells and that this is only corrected with FTI treatment, which correlates with the ability of chromosomes to be able to relocate rapidly. Furthermore, we indicate that lamin A is involved in chromosome positioning and behavior, which could be regulated via NM1β as part of a nuclear motor.

## Materials and methods

### Cell culture and treatments

Control human HDFs, 2DD [62], and HDFs derived from three classic HGPS patients (cell lines AG11513, AG01972C and AG11498, Coricell Repositories) were cultured in 15% FBS in DMEM with passaging twice every week. The proliferative status of the cell cultures was assessed by the presence of pKi-67 in cells [53] using indirect immunofluorescence. In 2DD HDFs the pKi-67 fraction of cells ranged from 40% to 20%. For HGPS cells the range was 70% to 2% over time in culture, demonstrating hyperproliferation in the HGPS cells, as has been determined before [63]. To elicit a chromosome movement response, cells were grown in 15% FBS for 2 days and then placed in 0.5% FBS in DMEM for 5 minutes, 10 minutes, 15 minutes, 30 minutes or 7 days. For serum restoration experiments the cells were cultured in 15% FBS in DMEM for 2 days, then placed in 0.5% FBS in DMEM for 7 days, which was replaced with 15% FBS in DMEM for 8 hours, 24 hours, 32 hours and 36 hours.

### Treatment with farnesyltransferase I and geranylgeranyltransferase inhibitors

Inhibitors of farnesylation and prenylation used in this study were FTI-277 (Calbiochem-Novabiochem, La Jolla, CA, USA) and GGTI-2147 (Calbiochem-Novabiochem). Both inhibitors were dissolved in DMSO and stored at -20°C. HGPS HDFs were seeded at 2 × 105 cells in 10 cm^2 ^tissue culture dishes and then allowed to grow for at least 2 days in 15% FBS in DMEM. Cells were incubated with 2.5 μM final concentration of FTI-277 and 2.5 μM of GGTI-2147 in 15% FBS in DMEM for 48 hours.

### Nuclear myosin inhibitor treatment

Myosin polymerization was inhibited by treating cells with 10 mM BDM (Calbiochem) for 15 minutes [42].

### Two-dimensional FISH

For the two-dimensional FISH assay, HDFs were harvested and placed in hypotonic buffer (0.075 M KCl, w/v) for 15 minutes at room temperature and spun at 400 g. The cells were fixed in 3:1 (v/v) methanol:acetic acid between five and seven times before being dropped onto humidified glass microscope slides. After dehydration in an ethanol row the cells were denatured in 70% formamide, 2× sodium saline citrate buffer (SSC), pH 7, at 70°C for 2 minutes. Chromosome paints for chromosomes 10, 18 and × were amplified from flow-sorted whole chromosome templates and labeled with biotin-16-dUTP by Degenerate OligoPrimer-PCR [64]. We used 200 to 400 μg chromosome paint, 7 μg C0*t-*1 DNA and 3 μg herring sperm per slide. Hybridization was performed in a humidified chamber for 18 to 24 hours at 37°C. The slides were washed in 50% formamide, 2× SSC, pH 7, at 45°C for 15 minutes, followed by 0.1× SSC prewarmed to 60°C for 15 minutes at 45°C. Labeled hybridized probes were detected with streptavidin-cyanine 3 (Amersham Life Science Ltd, GE Healthcare UK Ltd, Little Chalfont, Buckinghamshire, UK).

### Three-dimensional FISH

For the three-dimensional FISH assay, fibroblasts were washed in PBS and then fixed in 4% paraformaldehyde (w/v) in PBS for 10 minutes. A permeabilization step was performed with 0.5% Triton-X100 (v/v) and 0.5% saponin (w/v) in PBS for 20 minutes. The cells were then incubated in 20% glycerol in PBS for 30 minutes prior to being snap-frozen in liquid nitrogen. The cells were repeatedly frozen and thawed up to five times. After the freeze-thaw cycles, the cells were washed in PBS for at least 30 minutes and then incubated in 0.1 N HCl for 10 minutes. The cells were then washed in 2× SSC for 15 minutes and incubated in 50% formamide, 2× SSC, pH 7.0, overnight. For denaturation, cells were incubated at 73 to 76°C in 70% formamide, 2× SSC, pH 7, solution for 3 minutes and then were immediately transferred to 50% formamide, 2× SSC, pH 7, solution for 1 minute at the same temperature. All the subsequent steps were as for two-dimensional FISH.

### Indirect immunofluorescence

To reveal proliferating cells, rabbit anti-Ki-67 antibody (1:1,500; Dako, Ely, Cambridgeshire, UK). or mouse anti-pKi67 were incubated with the fixed cells. Secondary antibodies employed were swine anti-rabbit conjugated to either fluorescein isothiocynate (FITC; 1:30; Dako) or tetrarhodamine isothiocynate (TRITC; 1:30; Dako) and donkey anti-mouse conjugated to FITC. Rabbit anti-NM1β (1:200; Sigma-Aldrich, Poole, Dorset, UK) was used to reveal NM1β distribution with swine anti-rabbit conjugated to TRITC (Dako) as the secondary antibody.

### Image capture and analysis

For two-dimensional FISH analyses, digital grey-scale images of random nuclei were captured using a Photometrics cooled CCD camera on a Leica fluorescence microscope (Leitz DMRB) using a Plan Fluotar 100× oil immersion lens and Digital Scientific Smart Capture software. The images were run through a simple erosion script in IPLab spectrum software as described in [38]. DAPI images of the nucleus were outlined and divided into 5 concentric shells of equal area, the first shell being most peripheral and the innermost denoting the interior of the nucleus. The script measures the pixel intensity of DAPI and the chromosome probe in these five shells. The probe signal was normalized by dividing the percentage of the probe by the percentage of DAPI signal in each shell. Histograms were plotted with standard error bars representing standard error of the mean. Simple statistical analyses were performed using the unpaired two-tailed student's *t*-test using Microsoft excel.

For three-dimensional FISH analyses, images of nuclei were captured using a Nikon confocal laser scanning microscope (TE2000-S) equipped with a 60×/1.49 Nikon Apo oil immersion objective. The microscope was controlled by Nikon confocal microscope C1 (EZ-C1) software version 3.00. Stacks of optical sections with an axial distance of 0.2 μm were collected from 20 random nuclei. Stacks of 8-bit gray-scale two-dimensional images were obtained with a pixel dwell of 4.56 and eight averages were taken for each optical image. The positioning of chromosomes in relation to the nuclear periphery was assessed by performing measurements using Imaris Software (Bitplane Scientific Solutions, Zurich, CH-8048, Switzerland) whereby the distance in micrometers between the geometric center of each chromosome territory and the nearest nuclear periphery, as determined by DAPI staining, was measured in three dimensions. These data were not normalized for size but when the data were normalized by dividing by the length of the major axis plus the length of the minor axis divided by 2 or the length of the major axis alone, the relative positions of the individual chromosomes in frequency distributions did not change. Frequency distribution curves were plotted with the distance between the geometric center of chromosome territory and the nearest nuclear periphery on the x-axis in actual micrometers and the frequency on the y-axis.

## Abbreviations

BDM: 2,3-butanedione-2-monoxime; DAPI: 4',6-diamidino-2-phenylindole; DMEM: Dulbecco's modified Eagle's medium; DMSO: dimethyl sulfoxide; FBS: fetal bovine serum; FISH: fluorescence *in situ *hybridization; FITC: fluorescein isothiocynate;FTI: farnesyltransferase inhibitor; GGTI: geranylgeranyltransferase inhibitor; HDF: human dermal fibroblasts; HGPS: Hutchinson-Gilford progeria syndrome; NM1β: nuclear myosin 1β; PBS: phosphate-buffered saline; SSC: saline-sodium citrate; TRITC: tetrarhodamine isothiocyanate.

## Authors' contributions

ISM designed and performed the majority of the experimentation, gathered and analyzed data and drafted the manuscript. CHE helped to design one of the supplementary experiments, performed some laboratory work and helped write the manuscript. HDA performed some of the laboratory work. IRK helped design some of the experiments and aided in writing the manuscript. JMB designed the study and some of the experimentation, performed some laboratory work, acquired and analyzed data and wrote the final version of the manuscript. All authors saw and approved the final version of the manuscript.

## Supplementary Material

Additional file 1 **Supplementary Figures S1 and S2**. Figure S1: relative nuclear positions of chromosome 10 and × territories in proliferating HGPS fibroblasts determined using three-dimensional FISH. The positions of chromosome 10 and × territories in proliferating AG01972 HDFs (a cell line derived from a HGPS patient) were analyzed using a three-dimensional FISH assay. Cells were fixed using 4% paraformaldehyde to maintain the three-dimensional structure and then subjected to a three-dimensional FISH assay to delineate the area occupied by a particular chromosome territory. Stacks of optical sections with an axial distance of 0.2 μm were captured from at least 20 random nuclei. The distance between the geometric center of the chromosome territory and the nearest nuclear periphery was then measured. **(a, b) **The relative distances of chromosome 10 territories (a) and chromosome × territories (b) from the nearest nuclear periphery in proliferating HGPS fibroblasts (red line) are shown and compared to relative distances of chromosome 10 (a) and × (b) territories in control proliferating (blue dashed line), quiescent (green dashed line) and senescent (orange dashed line) HDFs. Figure S2: there is no active chromosome movement in FTI-treated HGPS cells after inhibition of nuclear myosin using BDM. The HGPS cell line AG11498 was grown in the presence of a FTI for 48 hours and was either left in 15% FBS (red bars), placed in 0.5% serum (blue bars) for 15 minutes or placed in low serum for 15 minutes with a 15 minute incubation in BDM to inhibit myosin activity (green bars). Cells were fixed for and subjected to two-dimensional FISH using a whole chromosome painting probe for chromosome 10. Images of pKi-67 positive cells were collected and analyzed by the bespoke erosion script [39]. A 48-hour FTI treatment restored the response to serum removal in HGPS cells, with chromosome 10 movement towards the nuclear periphery. This movement of chromosome 10 in FTI-treated HGPS cells was inhibited by treatment with BDM, which affects the polymerization and activity of nuclear myosin. Click here for file
