# Supplementary material for: Farnesyltransferase inhibitor treatment restores chromosome territory positions and active chromosome dynamics in Hutchinson-Gilford progeria syndrome cells
Source: Genome Biol. 2011 Aug 12;12(8):R74. doi: 10.1186/gb-2011-12-8-r74 (PMC3245614; doi:10.1186/gb-2011-12-8-r74)
Supplement: Additional file 1 — Supplementary Figures S1 and S2. Figure S1: relative nuclear positions of chromosome 10 and × territories in proliferating HGPS fibroblasts determined using three-dimensional FISH. The positions of chromosome 10 and × territories in proliferating AG01972 HDFs (a cell line derived from a HGPS patient) were analyzed using a three-dimensional FISH assay. Cells were fixed using 4% paraformaldehyde to maintain the three-dimensional structure and then subjected to a three-dimensional FISH assay to delineate the area occupied by a particular chromosome territory. Stacks of optical sections with an axial distance of 0.2 μm were captured from at least 20 random nuclei. The distance between the geometric center of the chromosome territory and the nearest nuclear periphery was then measured. (a, b) The relative distances of chromosome 10 territories (a) and chromosome × territories (b) from the nearest nuclear periphery in proliferating HGPS fibroblasts (red line) are shown and compared to relative distances of chromosome 10 (a) and × (b) territories in control proliferating (blue dashed line), quiescent (green dashed line) and senescent (orange dashed line) HDFs. Figure S2: there is no active chromosome movement in FTI-treated HGPS cells after inhibition of nuclear myosin using BDM. The HGPS cell line AG11498 was grown in the presence of a FTI for 48 hours and was either left in 15% FBS (red bars), placed in 0.5% serum (blue bars) for 15 minutes or placed in low serum for 15 minutes with a 15 minute incubation in BDM to inhibit myosin activity (green bars). Cells were fixed for and subjected to two-dimensional FISH using a whole chromosome painting probe for chromosome 10. Images of pKi-67 positive cells were collected and analyzed by the bespoke erosion script [39]. A 48-hour FTI treatment restored the response to serum removal in HGPS cells, with chromosome 10 movement towards the nuclear periphery. This movement of chromosome 10 in FTI-treated HGPS cells was inhibited by t [file gb-2011-12-8-r74-S1.PPTX]

## Slide 1
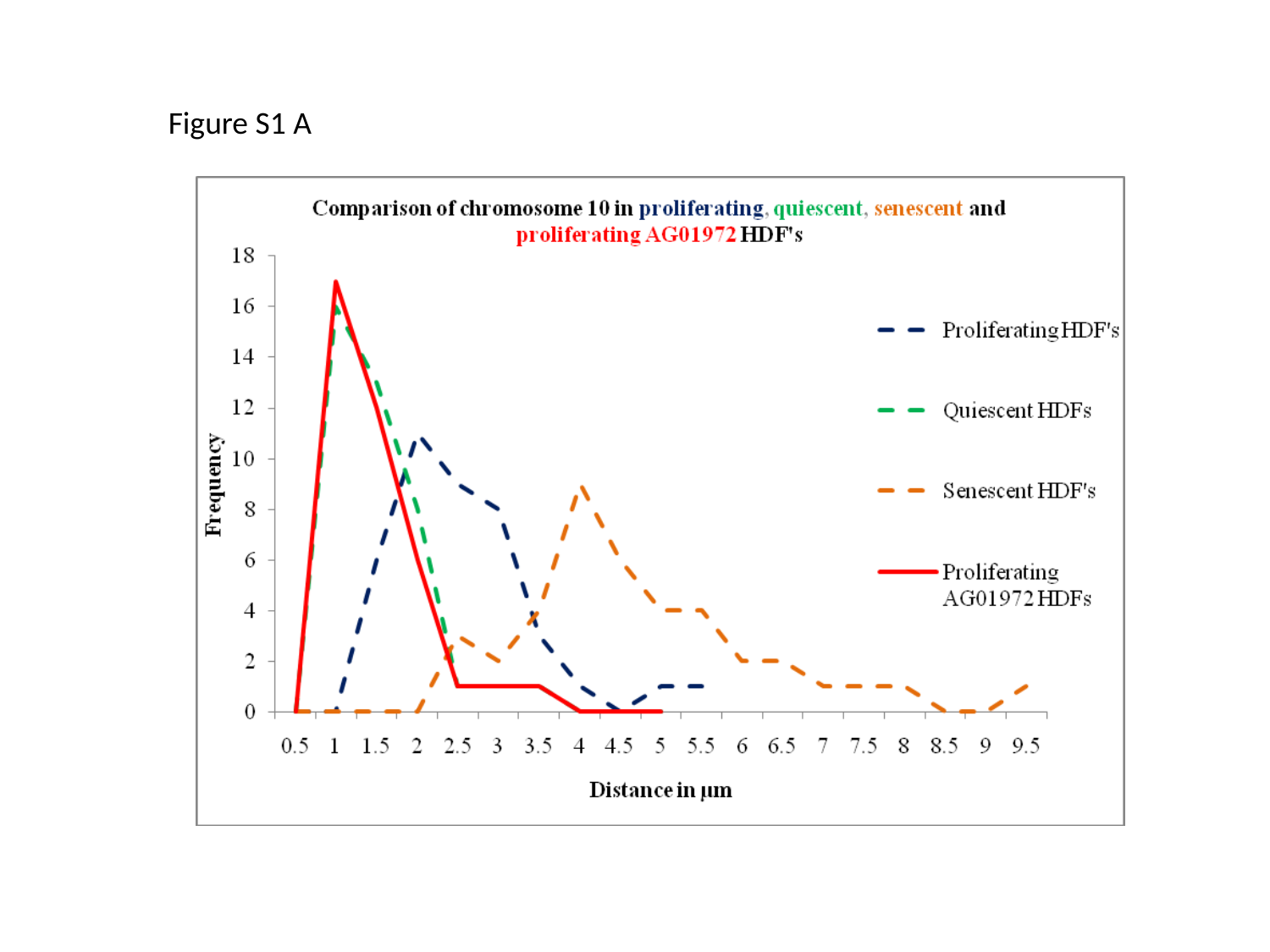

Figure S1 A

## Slide 2
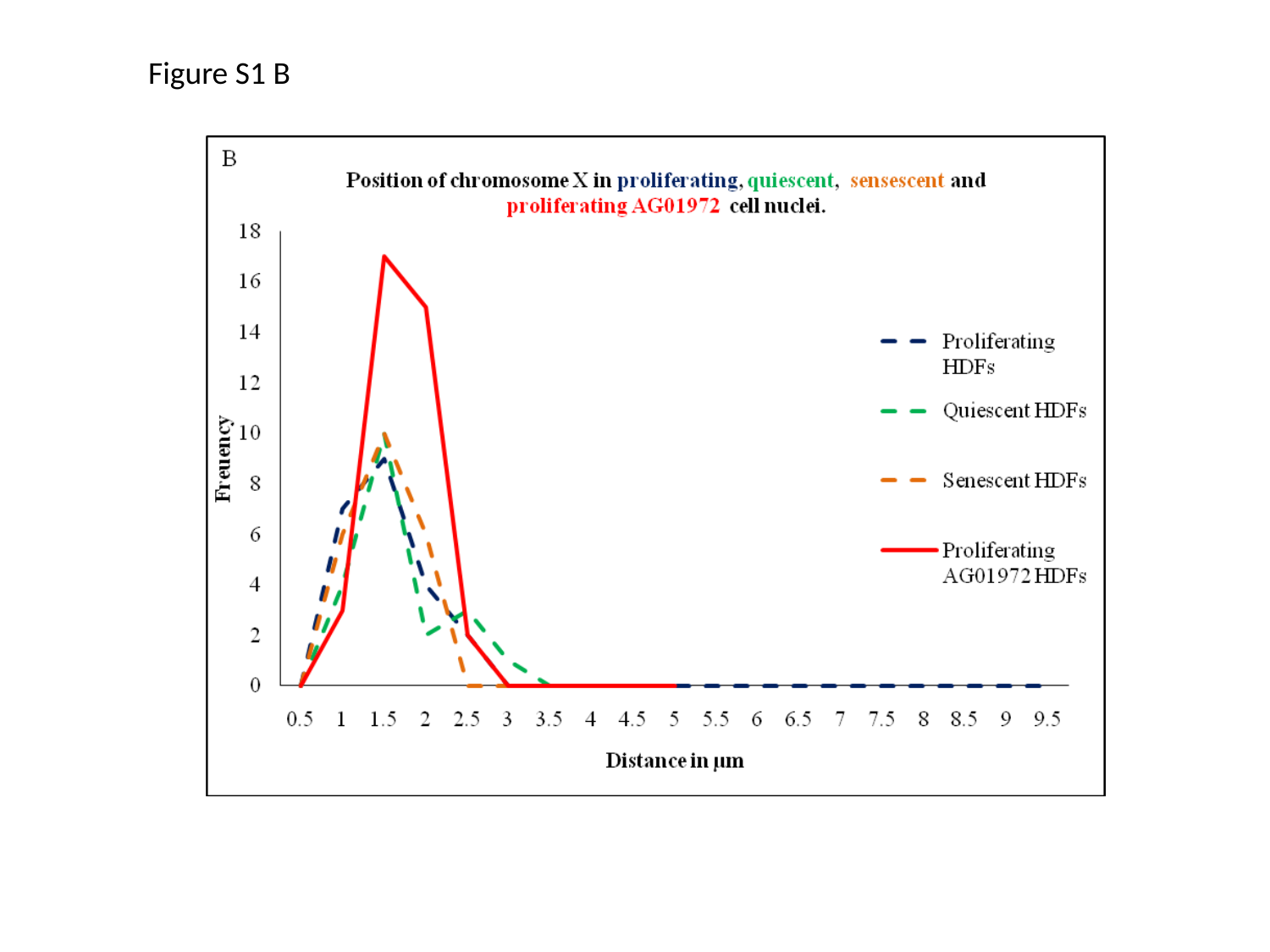

Figure S1 B

## Slide 3
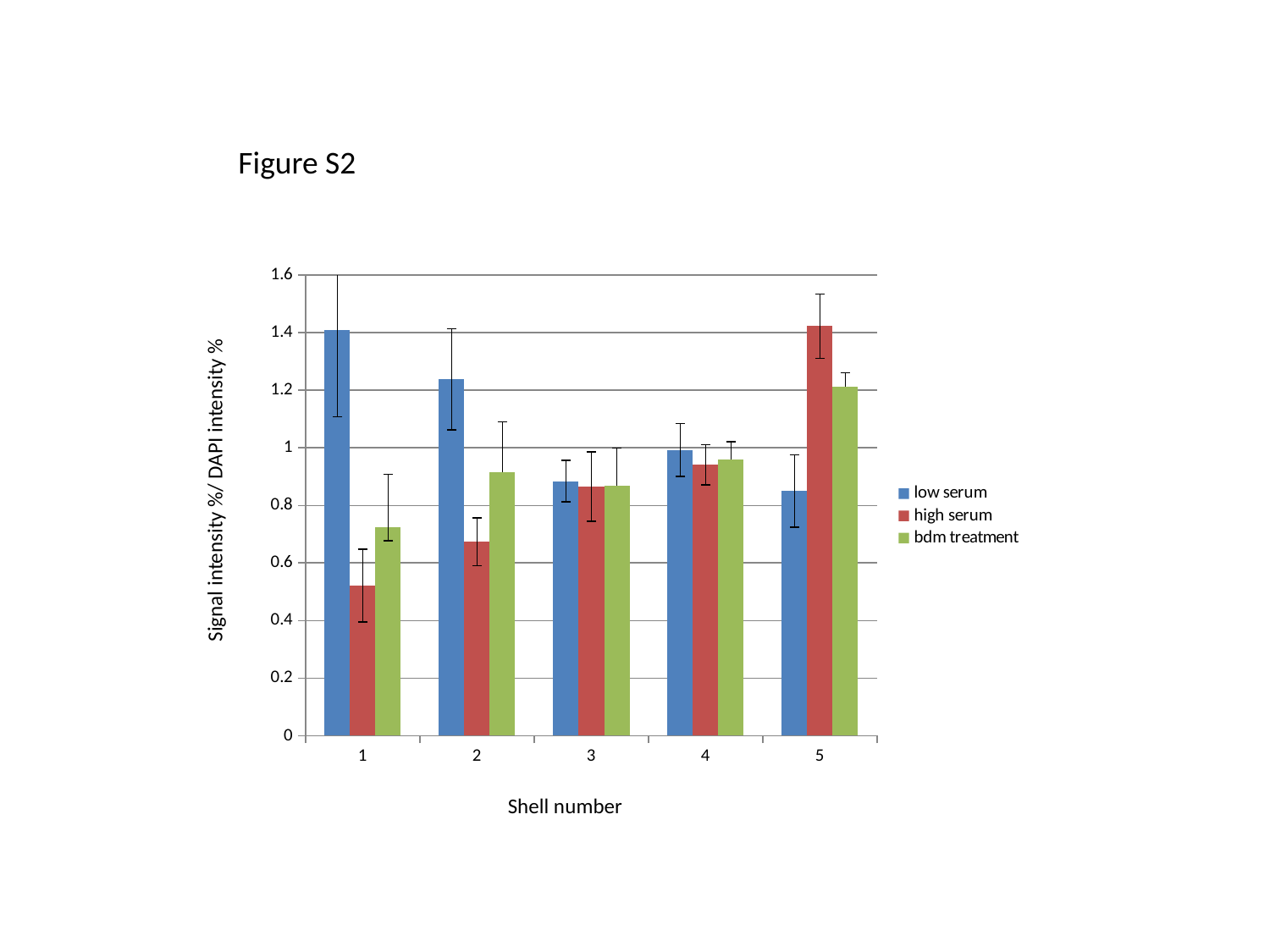

Figure S2
### Chart
| Category | low serum | high serum | bdm treatment |
|---|---|---|---|Signal intensity %/ DAPI intensity %
Shell number
